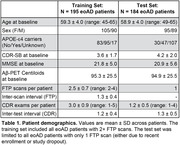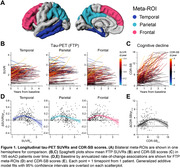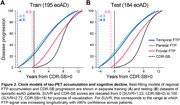# A clock model of regional tau‐PET accumulation and cognitive decline in sporadic early‐onset Alzheimer’s disease

**DOI:** 10.1002/alz.091032

**Published:** 2025-01-09

**Authors:** Daniel R. Schonhaut, Philip S. Insel, David N. soleimani‐meigooni, Ehud Zeltzer, Charles Windon, Piyush Maiti, Claire Yballa, Jiaxiuxiu Zhang, Nidhi S Mundada, Ranjani Shankar, Alinda Amuiri, Robert A. Koeppe, Bradford C. Dickerson, Maria C. Carrillo, Liana G. Apostolova, Gil D. Rabinovici, Renaud La Joie

**Affiliations:** ^1^ Memory and Aging Center, Weill Institute for Neurosciences, University of California, San Francisco, San Francisco, CA USA; ^2^ Department of Epidemiology & Biostatistics, University of California, San Francisco, San Francisco, CA USA; ^3^ University of Michigan, Ann Arbor, MI USA; ^4^ Massachusetts Alzheimer’s Disease Research Center, Massachusetts General Hospital, Boston, MA USA; ^5^ Alzheimer's Association, Chicago, IL USA; ^6^ Indiana Alzheimer's Disease Research Center, Indianapolis, IN USA

## Abstract

**Background:**

The timing of tau‐PET accumulation and cognitive decline in sporadic early‐onset Alzheimer’s disease (eoAD, age‐at‐onset<65) has not been established and is needed to optimize tau‐PET as an outcome measure in clinical trials. Here we leverage large‐sample, longitudinal data from the Longitudinal Early‐onset Alzheimer’s Disease Study (LEADS) to model tau‐PET accumulation in three regions relative to cognitive decline.

**Method:**

Longitudinal [^18^F]Flortaucipir‐PET (FTP) and CDR‐SB scores were acquired in 195 amyloid‐PET‐positive, sporadic eoAD patients with MCI or mild dementia due to AD at baseline (Table 1). FTP scans were referenced against inferior cerebellar GM, and mean SUVRs calculated in three meta‐ROIs comprising temporal, parietal, and frontal association cortices (Figure 1A). Generalized additive models estimated nonlinear relations between: (1) baseline SUVRs and annualized rates‐of‐change in each meta‐ROI, and (2) baseline CDR‐SB and annualized rates‐of‐change in cognition. Integrating these baseline‐by‐change functions yielded regional FTP and CDR‐SB trajectories over time. An exhaustive search algorithm identified optimal time‐shifts between each regional tau trajectory and CDR‐SB progression that minimized the error between model‐predicted and observed data at baseline. This time‐shifting procedure was repeated in a held‐out dataset of 184 eoAD patients to assess out‐of‐sample reproducibility (Table 1).

**Result:**

FTP SUVRs and CDR‐SB scores increased longitudinally in most patients (Figure 1B,C). Baseline SUVR by rate‐of‐change associations were inverted U‐shaped in each meta‐ROI (Figure 1D), and similar associations were found for CDR‐SB progression (Figure 1E). Time‐shift estimation in the training dataset converged on a single, best‐fitting model in which temporal and parietal FTP accumulation led clinical onset by 4.5 years, while frontal FTP accumulation began 0.5 years before clinical onset (Figure 2A). The replication dataset yielded nearly identical results (Figure 2B). We estimate that the full timecourse of FTP accumulation and cognitive decline occurs over ∼16 years from earliest detectable change in FTP.

**Conclusion:**

Tau‐PET accumulation in the frontal meta‐ROI immediately precedes cognitive decline and tracks closely with clinical progression in eoAD, while temporoparietal tau‐PET is prominent at clinical onset and plateaus as dementia worsens. For clinical trials in symptomatic patients, temporal and parietal tau‐PET may therefore aid staging and inclusion criteria, while frontal tau‐PET is likely superior for monitoring longitudinal change.